# Supplementary material for: Bactericidal antibiotic treatment induces damaging inflammation via TLR9 sensing of bacterial DNA
Source: Nat Commun. 2024 Nov 28;15:10359. doi: 10.1038/s41467-024-54497-3 (PMC11605096; doi:10.1038/s41467-024-54497-3)
Supplement: Supplementary file 1 — Supplementary Information [file 41467_2024_54497_MOESM1_ESM.pdf]

Supplementary Information

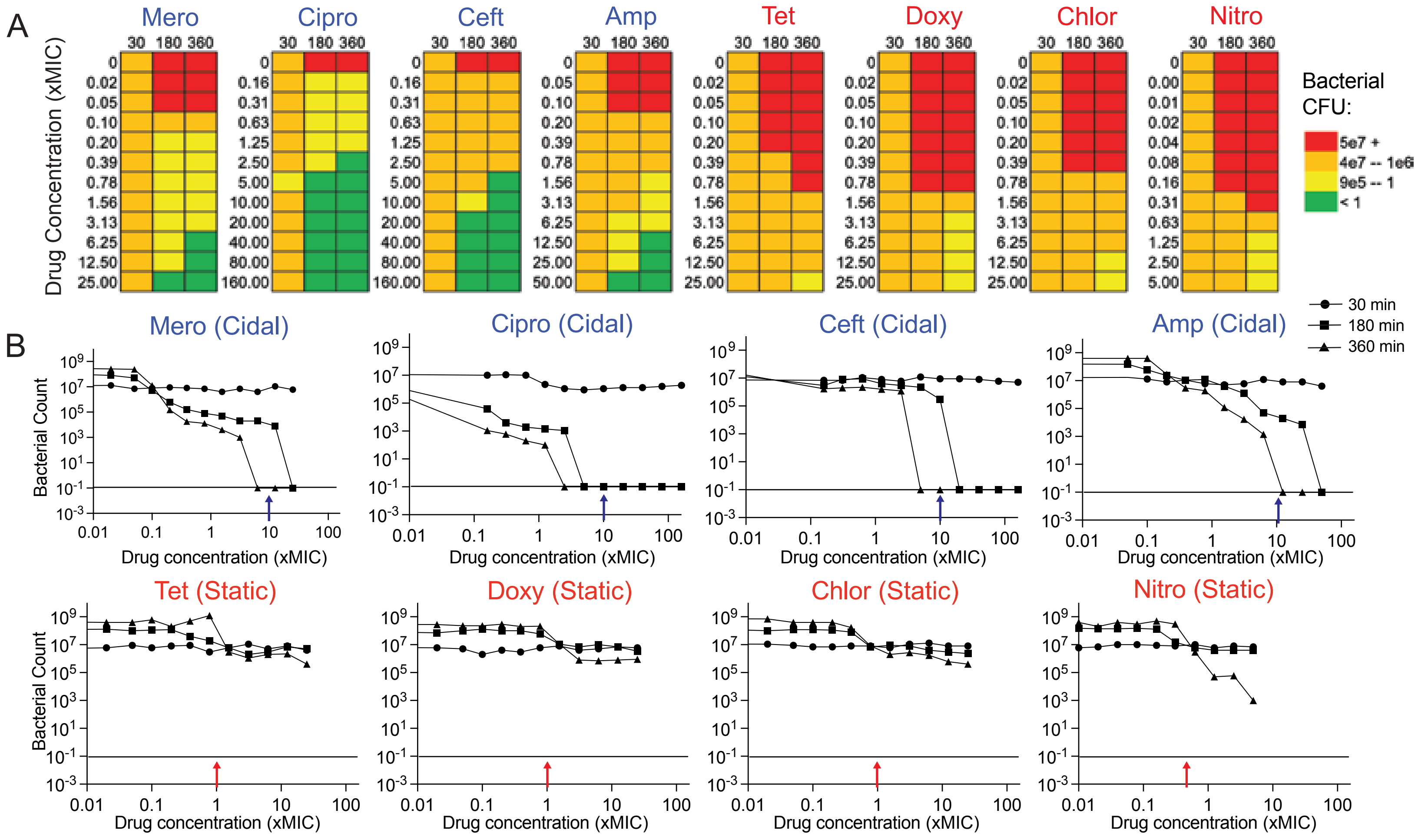
A
Mero
Cipro
Ceft
Amp
Tet
Doxy
Chlor
Nitro
30 180 360
Drug Concentration (xMIC)
Bacterial CFU:
5e7 +
4e7 -- 1e6
9e5 -- 1
< 1
B
Mero (Cidal)
Cipro (Cidal)
Ceft (Cidal)
Amp (Cidal)
30 min
180 min
360 min
Tet (Static)
Doxy (Static)
Chlor (Static)
Nitro (Static)
Bacterial Count
Drug concentration (xMIC)

Figure S1

Figure S1. Antibiotic Concentrations and Exposure Intervals that Cause Killing (cidal drugs) and Growth Arrest (static drugs). (A-B) Colony forming unit (CFU) quantifications of bacteria treated with varying concentrations of antibiotics for 30, 180, and 360 minutes. Concentrations are expressed in terms of drug-specific MIC, see Table 1. Horizontal lines at 10^{-1} in panel B denote the limit of detection (0 bacteria counted). Each quantitation is representative of single measurements in 2-3 independent experiments. Source data are provided as a Source Data file.

A

Tetracycline

Drug Concentration (xMIC)

MOI	0	-25	-5	1	5
0.08	1.00	1.59	0.50	0.18	0.06
0.4	1.00	1.44	0.74	0.29	0.18
2	1.00	1.17	1.09	0.39	0.32
10	1.00	1.18	2.52	0.59	0.53

Chloramphenicol

Drug Concentration (xMIC)

MOI	0	-25	-5	1	5
0.08	1.00	1.06	0.86	0.64	0.78
0.4	1.00	1.53	0.84	0.35	0.39
2	1.00	1.24	1.78	1.29	1.17
10	1.00	1.49	2.17	2.36	2.01

0 1 2 3

Fold Change (normalized to untreated)

B

Meropenem

Drug Concentration (xMIC)

MOI	0	-25	-5	1	5
0.08	1.00	1.00	1.10	1.16	1.52
0.4	1.00	1.08	1.03	1.91	2.10
2	1.00	1.15	1.44	2.37	2.51
10	1.00	1.93	2.51	2.71	2.69

Ampicillin

Drug Concentration (xMIC)

MOI	0	-25	-5	1	5
0.08	1.00	1.27	1.36	1.32	1.39
0.4	1.00	1.27	2.10	1.88	1.85
2	1.00	1.56	2.06	2.49	2.33
10	1.00	1.40	1.73	3.07	2.78

Figure S2

Figure S2. Extended K12 Antibiotic Screening Data. (A-B) TNF quantified by ELISA at 8hr from RAW macrophages infected with static **(A)** or cidal **(B)** drug treated K12 *E. coli* at a range of MOIs (moieties of infection), and antibiotic concentrations of the indicated antibiotic. Data is presented as fold change relative to the uninfected bacteria at the indicated MOI and colored on a low (red) – high (green) scale. Each quantitation is representative of three independent measurements in 2-3 independent experiments. Source data are provided as a Source Data file.

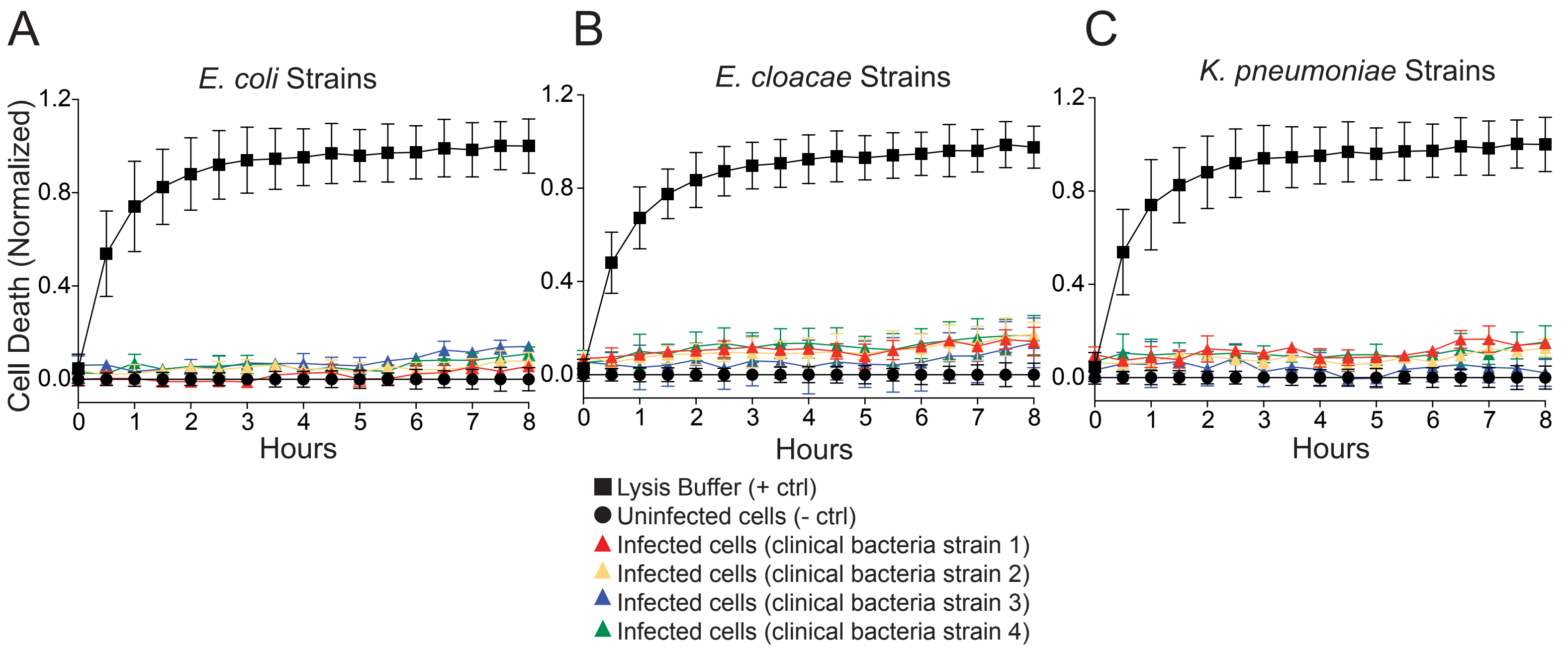
A
E. coli Strains
B
E. cloacae Strains
C
K. pneumoniae Strains
Cell Death (Normalized)
1.2
0.8
0.4
0.0
0
1
2
3
4
5
6
7
8
Hours
Lysis Buffer (+ ctrl)
Uninfected cells (- ctrl)
Infected cells (clinical bacteria strain 1)
Infected cells (clinical bacteria strain 2)
Infected cells (clinical bacteria strain 3)
Infected cells (clinical bacteria strain 4)

Figure S3

Figure S3. Macrophage Viability is Maintained During Bacterial Infections. (A-C) iBMDM cell death quantified over an 8-hr time course for cells challenged with lysis buffer (positive control), media alone (negative control), or each of 4 clinical isolates at 10x MOI of *E. coli*, **(A)** *E. cloace*, **(B)** or *K. pneumoniae* **(C)** as indicated in the individual graphs. All cells were incubated with media containing CellTox Green and death was quantified in triplicate via fluorescence, with one of two representative experiments shown. Error bars display SEM. Source data are provided as a Source Data file.

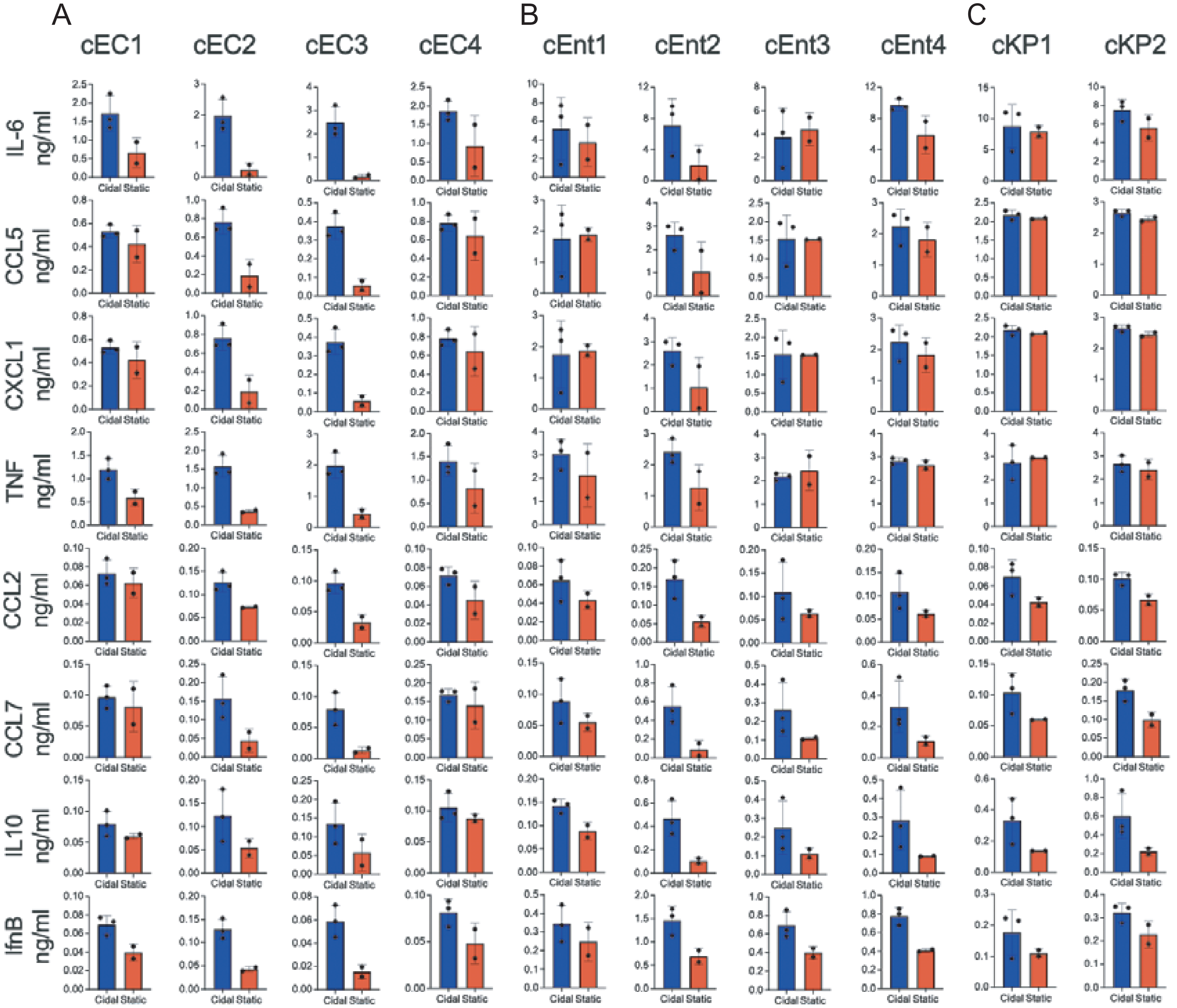
A
B
C
cEC1
cEC2
cEC3
cEC4
cEnt1
cEnt2
cEnt3
cEnt4
cKP1
cKP2
IL-6 ng/ml
CCL5 ng/ml
CXCL1 ng/ml
TNF ng/ml
CCL2 ng/ml
CCL7 ng/ml
IL10 ng/ml
IfnB ng/ml
Cidal Static

Figure S4

Figure S4. Cidal antibiotic treatments induce more proinflammatory cytokines pattern preserved across clinical strains. (A-C) Eight cytokine outputs were quantified via Luminex from iBMDMs infected at 10x MOI with a panel of 4 *E. coli* **(A)**, *E. cloace*, **(B)** or *K. pneumoniae* **(C)** isolates treated with various antibiotics for 6.5 hrs. These cytokines were selected from a broader screen and were the only ones in which we saw consistent responses above the LOD. Results are plotted as the average of three cidal treated infections (mero, cipro, and ceft) and two satic treated infections (doxy, nitro) for each clinical strain, and the indicated cytokine readout. **(A-C)** Results are of a single large screening experiment. Error bars display SEM. Source data are provided as a Source Data file.

A

MOI 10	cEC1	cEC2	cEC3	cEC4	cEnt1	cEnt2	cEnt3	cEnt4	cKP1	cKP3
IL6	158%	716%	1342%	101%	38%	257%	-15%	65%	11%	34%
CCL5	25%	306%	557%	21%	-7%	150%	1%	23%	4%	8%
CXCL1	68%	219%	411%	38%	-5%	129%	7%	33%	-35%	4%
TNF	103%	320%	358%	71%	43%	91%	-11%	7%	-7%	10%
CCL2	17%	74%	191%	59%	49%	201%	71%	77%	64%	52%
CCL7	19%	253%	499%	20%	62%	522%	141%	203%	74%	81%
IL10	33%	129%	133%	19%	61%	346%	125%	212%	141%	171%
InfB	75%	200%	284%	70%	38%	111%	73%	90%	60%	41%

MOI 2	cEC1	cEC2	cEC3	cEC4	cEnt1	cEnt2	cEnt3	cEnt4	cKP1	cKP3
IL6	220%	516%	98%	115%	24%	381%	67%	104%	-13%	15%
CCL5	47%	176%	312%	35%	28%	183%	23%	53%	-1%	8%
CXCL1	55%	130%	283%	31%	2%	122%	50%	21%	-62%	-21%
TNF	78%	211%	278%	50%	33%	117%	25%	12%	-20%	8%
CCL2	58%	61%	153%	21%	71%	169%	105%	119%	98%	53%
CCL7	81%	114%	251%	1%	100%	471%	187%	293%	95%	106%
IL10	37%	73%	112%	12%	37%	274%	121%	224%	170%	136%
InfB	97%	184%	388%	101%	33%	81%	92%	113%	31%	54%

MOI 0.4	cEC1	cEC2	cEC3	cEC4	cEnt1	cEnt2	cEnt3	cEnt4	cKP1	cKP3
IL6	101%	331%	480%	88%	6%	258%	71%	132%	10%	12%
CCL5	48%	122%	205%	65%	9%	144%	9%	45%	7%	33%
CXCL1	31%	54%	99%	16%	-16%	43%	34%	14%	-61%	-20%
TNF	42%	108%	161%	20%	3%	47%	14%	11%	-15%	10%
CCL2	71%	56%	98%	95%	138%	126%	81%	115%	164%	60%
CCL7	69%	14%	56%	88%	125%	300%	124%	189%	125%	109%
IL10	44%	59%	93%	39%	29%	149%	66%	172%	188%	47%
InfB	84%	110%	378%	91%	31%	77%	57%	104%	67%	35%

MOI 0.08	cEC1	cEC2	cEC3	cEC4	cEnt1	cEnt2	cEnt3	cEnt4	cKP1	cKP3
IL6	83%	218%	610%	46%	50%	171%	28%	145%	115%	45%
CCL5	89%	116%	376%	89%	27%	67%	-1%	77%	44%	40%
CXCL1	45%	44%	197%	13%	-8%	-18%	-11%	23%	29%	15%
TNF	52%	66%	258%	4%	-11%	14%	-7%	15%	12%	38%
CCL2	79%	50%	281%	92%	∞	117%	69%	214%	197%	264%
CCL7	95%	26%	227%	31%	227%	153%	89%	190%	131%	36%
IL10	91%	42%	213%	58%	23%	71%	8%	138%	220%	1258%
InfB	83%	218%	610%	46%	50%	171%	28%	145%	115%	45%

B

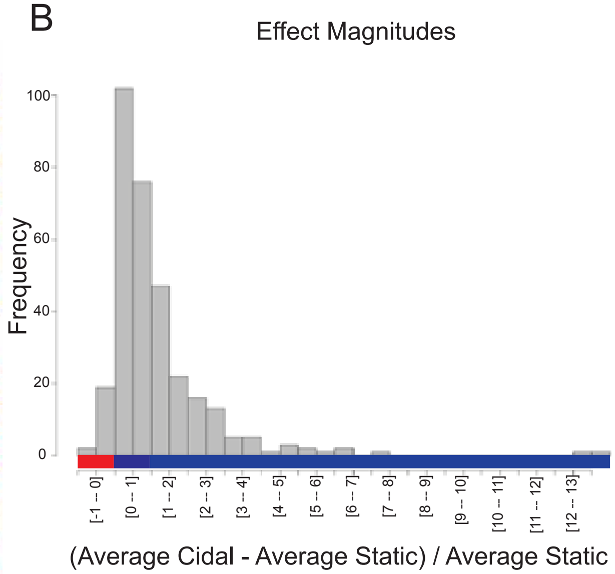

Figure S5

Figure S5. Full Luminex Screening. (A) Eight cytokine outputs were quantified via Luminex from iBMDMs infected at 10x MOI with a panel of 4 *E. coli*, *E. cloace*, or *K. pneumoniae* isolates treated with various antibiotics for 6.5 hrs across a variety of MOIs ranging from 10x – 0.08x. These cytokines were selected from a broader screen and were the only ones in which we saw consistent responses above the LOD. Results are plotted as (average cidal – average static) / average static for a given strain, cytokine, and MOI. Positive values (indicating the cidal average > the static average) are in blue, and negative values (indicating the cidal average < the static average) are in red. **(B)** Histogram of all 320 effect sizes of unique cidal vs. static comparisons (individual strain, readout, and MOI) across the entire Luminex run. X-axis color bar indicates static > cidal (red) vs. cidal > static (blue). **(A-B)** Results are of a single large screening experiment. Source data are provided as a Source Data file.

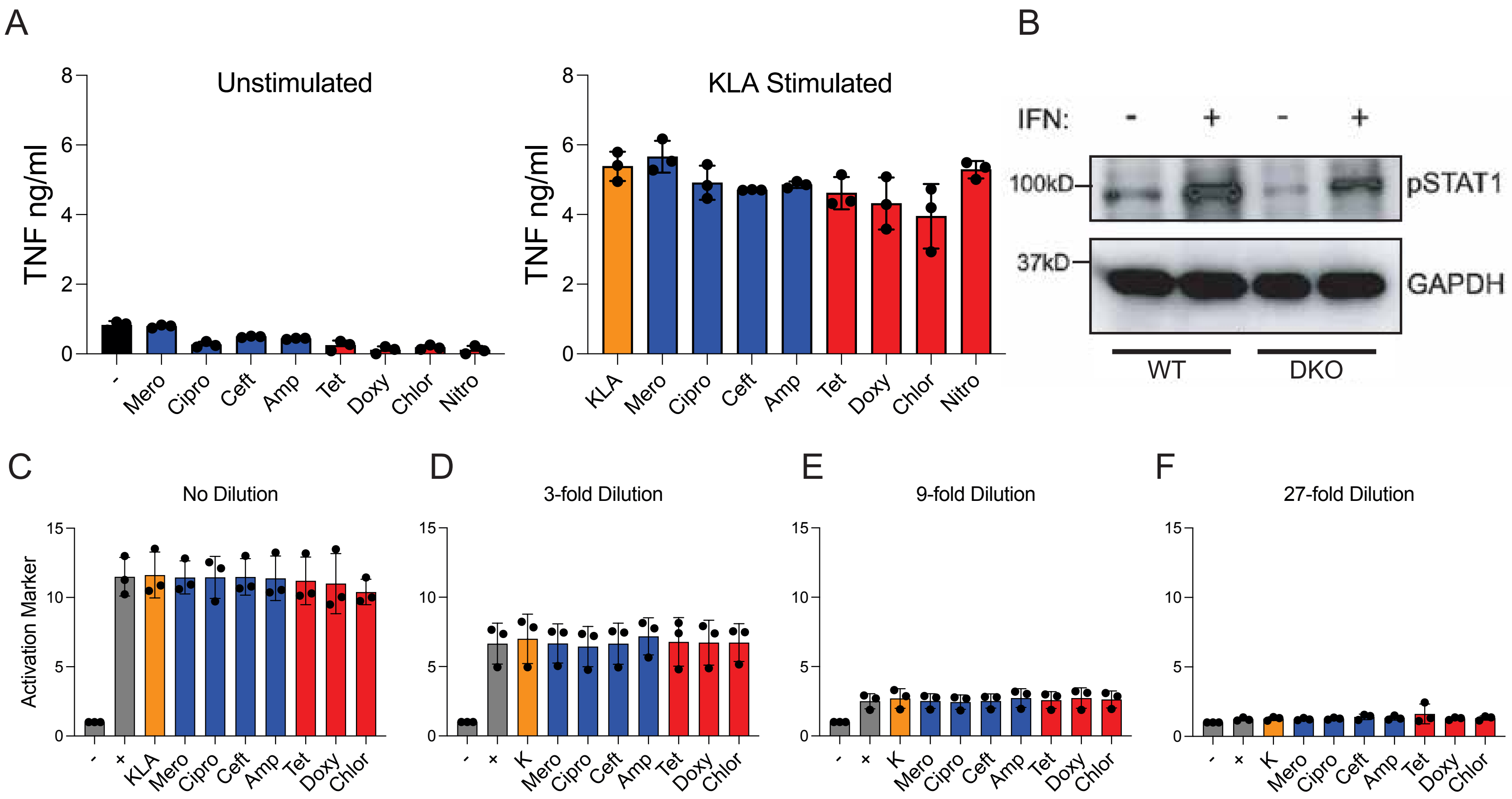
A
Unstimulated
TNF ng/ml
0
2
4
6
8
-
Mero
Cipro
Ceft
Amp
Tet
Doxy
Chlor
Nitro
KLA Stimulated
KLA
B
IFN:
-
+
100kD
37kD
pSTAT1
GAPDH
WT
DKO
C
No Dilution
Activation Marker
0
5
10
15
+
D
3-fold Dilution
K
E
9-fold Dilution
F
27-fold Dilution

Figure S6

Figure S6. Cell comparison internal controls. (A) TNF quantified by ELISA at 6.5 hr from iBMDMs that were incubated with each individual antibiotic at the concentrations we used for our cell comparisons in the absence of bacteria with no KLA **(**left graph), and with KLA (right graph). One representative experiment is shown of 3 independent experiments (3 technical replicates were run per condition per experiment). Error bars display SEM. **(B)** WT and STING KO iBMDMs were stimulated with mouse interferon beta for 30 min. pSTAT1 protein expression was quantified in each cell type by Western blot both with and without interferon. **(C-F)** WT iBMDM macrophages were infected with media alone (black), bacteria without antibiotics (black), KLA (orange), and equivalent concentrations of cidal (blue) and static treated bacteria (red) as indicated. Resulting supernatants were collected and applied to TLR4 reporter HEK cells: undiluted **(C)**, 3-fold diluted **(D)**, 9-fold diluted **(E)**, and 27-fold diluted **(F)**, and Quanti-blue detector reagent response was quantified. Error bars display SEM. **(B-F)** Results are representative of 2 independent experiments, with 3 technical replicates per condition. Source data are provided as a Source Data file.

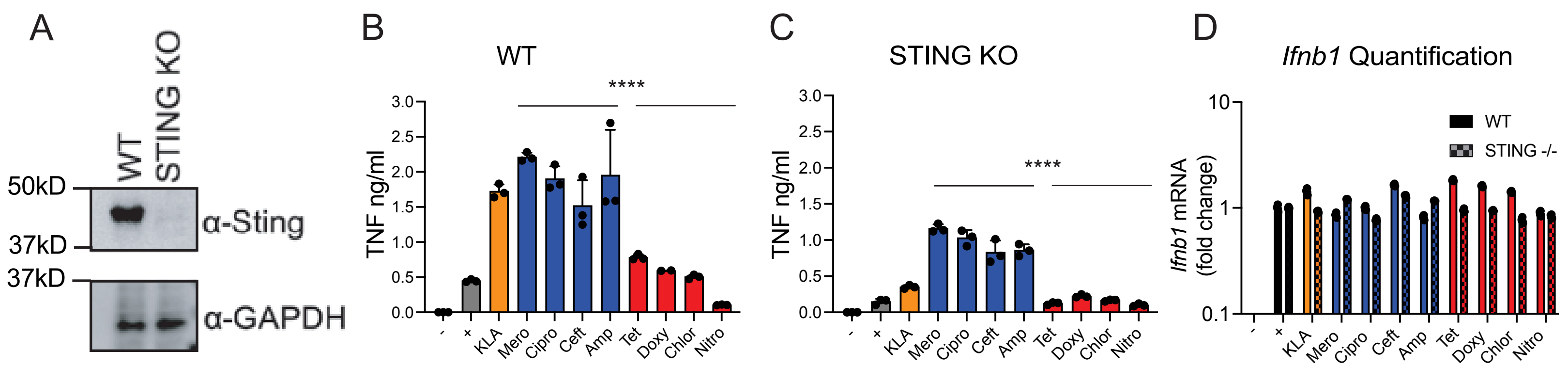
A
WT
STING KO
50kD
37kD
α-Sting
37kD
α-GAPDH
B
WT
TNF ng/ml
****
- + KLA Mero Cipro Ceft Amp Tet Doxy Chlor Nitro
C
STING KO
TNF ng/ml
****
- + KLA Mero Cipro Ceft Amp Tet Doxy Chlor Nitro
D
Ifnb1 Quantification
Ifnb1 mRNA (fold change)
WT
STING -/-
- + KLA Mero Cipro Ceft Amp Tet Doxy Chlor Nitro

Figure S7

Figure S7. STING is not responsible for cidal drug treatment mediated increases in inflammatory markers. (A) STING protein expression quantified by Western blot in WT and STING KO iBMDMs. **(B-C)** TNF quantified by ELISA at 6.5 hr from WT **(B)** and STING -/- iBMDM **(C)** macrophages infected with media alone (black), bacteria without antibiotics (black), KLA (orange), and equivalent concentrations of cidal (blue) and static treated bacteria (red) as indicated. Error bars display SEM. **(D)** Fold change in interferon beta mRNA from infections of WT or STING -/- iBMDMs with the indicated antibiotics quantified by qPCR. Results are shown on a log-10 scale. Error bars display SEM. **(A-D)** are representative of three independent experiments assessing three samples/group, and $*P < 0.05$, $**P < 0.01$, $***P < 0.001$, $****P < 0.0001$ by two-tailed t test. Source data are provided as a Source Data file.

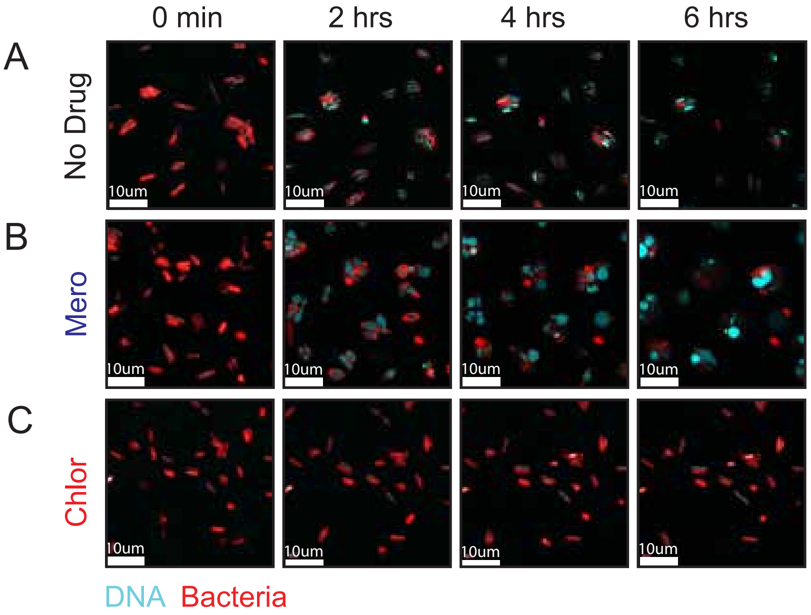

Figure S8

Figure S8. Live Imaging of Antibiotic Treated Bacteria. (A-C) Live imaging of untreated **(A)** mero-treated **(B)** or chlor-treated **(C)** Alexa 647-labeled bacteria (red) stained with Hoechst (cyan) for up to 6 hrs. One representative image is shown at each indicated timepoint (0hr, 2hr, 4hr, and 6hr). See Sup Movies 1-3 for accompanying videos of each time-course. **(A-C)** Results are representative of three independent experiments. Source data are provided as a Source Data file.

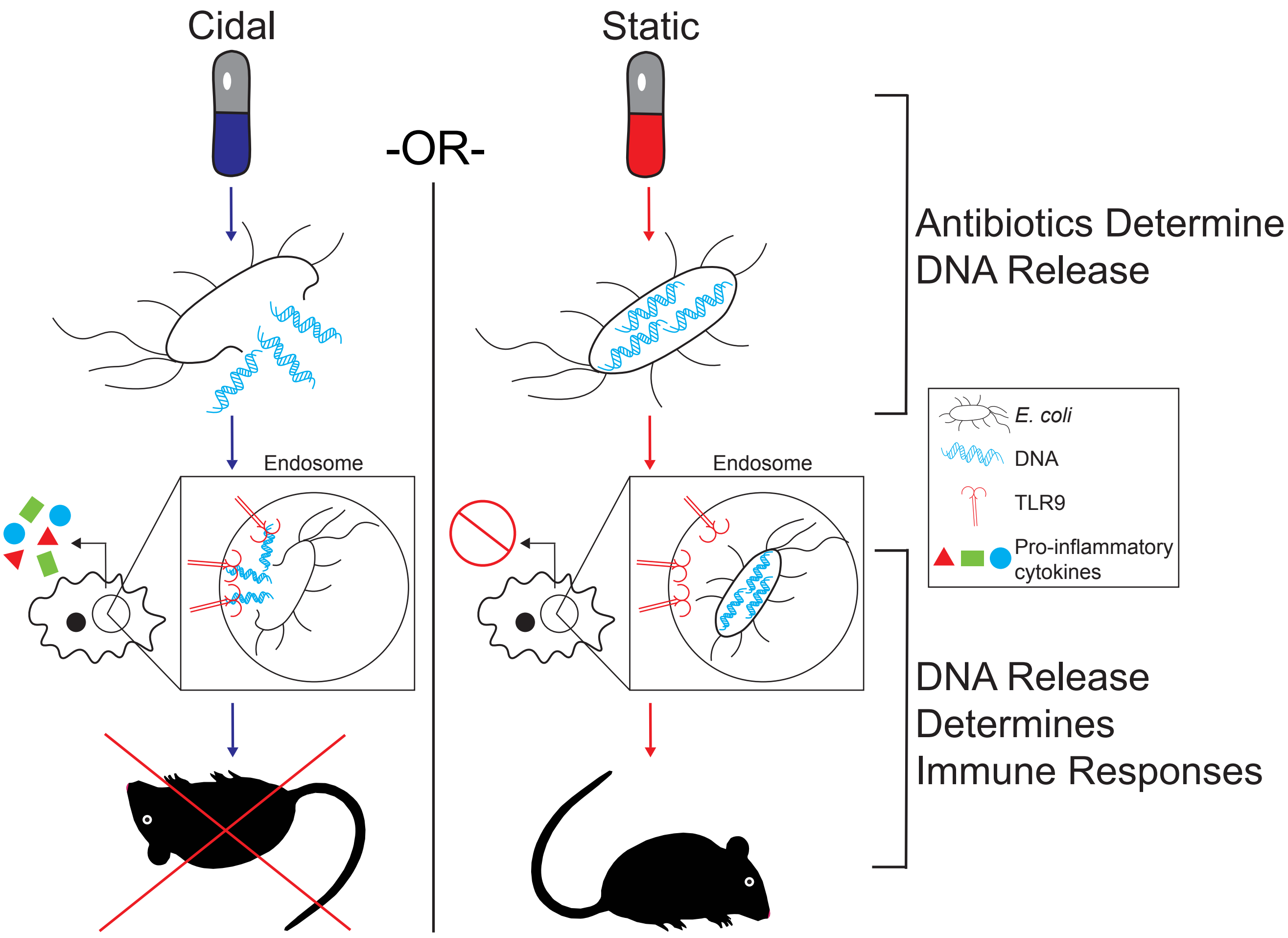
Cidal
-OR-
Static
Antibiotics Determine
DNA Release
Endosome
Endosome
E. coli
DNA
TLR9
Pro-inflammatory
cytokines
DNA Release
Determines
Immune Responses

Figure S9

Figure S9. Overall model. Cidal antibiotic treatment causes bacteria to release DNA (top left). Static antibiotic treatment does not cause bacteria to release DNA (top right). The presence (cidal drugs, right) or absence (static drugs, left) of DNA is sensed by TLR9 in macrophage endosomes, and converted into a strong pro-inflammatory cytokine response only in the presence of bacterial DNA. This extra inflammation, that occurs only when cidal drug released DNA is sensed, causes treatment failure in a murine peritonitis infection model (bottom left), while infected mice treated with the same dose of static drug are largely protected (bottom right). Cidal antibiotic efficacy is rescued in TLR9 deficient mice. Source data are provided as a Source Data file.
