# Supplementary material for: Bactericidal antibiotic treatment induces damaging inflammation via TLR9 sensing of bacterial DNA
Source: Nat Commun. 2024 Nov 28;15:10359. doi: 10.1038/s41467-024-54497-3 (PMC11605096; doi:10.1038/s41467-024-54497-3)
Supplement: Supplementary file 2 — Description of Additional Supplementary Files [file 41467_2024_54497_MOESM2_ESM.pdf]

Description of Additional Supplementary Files

Supplementary Movie 1-3. Time Course Live Imaging of Antibiotic Treated Bacteria.
Live imaging of untreated **(1)**, mero-treated **(2)**, or chlor-treated **(3)** Alexa 647- NHS ester-based labeling of cEC1 bacteria outer membrane proteins (red) stained with Hoechst (cyan) from 0-6 hrs. **(1-3)** Results are representative of three independent experiments.
