# Supplementary material for: Bactericidal antibiotic treatment induces damaging inflammation via TLR9 sensing of bacterial DNA
Source: Nat Commun. 2024 Nov 28;15:10359. doi: 10.1038/s41467-024-54497-3 (PMC11605096; doi:10.1038/s41467-024-54497-3)
Supplement: Supplementary file 6 — Reporting Summary [file 41467_2024_54497_MOESM6_ESM.pdf]

nature portfolio

Corresponding author(s): Iain Fraser and David Weiss

Last updated by author(s): Nov 1, 2024

Reporting Summary

Nature Portfolio wishes to improve the reproducibility of the work that we publish. This form provides structure for consistency and transparency in reporting. For further information on Nature Portfolio policies, see our Editorial Policies and the Editorial Policy Checklist.

Statistics

For all statistical analyses, confirm that the following items are present in the figure legend, table legend, main text, or Methods section.

n/a	Confirmed	
☐	☒	The exact sample size (*n*) for each experimental group/condition, given as a discrete number and unit of measurement
☐	☒	A statement on whether measurements were taken from distinct samples or whether the same sample was measured repeatedly
☐	☒	The statistical test(s) used AND whether they are one- or two-sided<br>*Only common tests should be described solely by name; describe more complex techniques in the Methods section.*
☐	☒	A description of all covariates tested
☐	☒	A description of any assumptions or corrections, such as tests of normality and adjustment for multiple comparisons
☐	☒	A full description of the statistical parameters including central tendency (e.g. means) or other basic estimates (e.g. regression coefficient) AND variation (e.g. standard deviation) or associated estimates of uncertainty (e.g. confidence intervals)
☐	☒	For null hypothesis testing, the test statistic (e.g. *F*, *t*, *r*) with confidence intervals, effect sizes, degrees of freedom and *P* value noted<br>*Give P values as exact values whenever suitable.*
☒	☐	For Bayesian analysis, information on the choice of priors and Markov chain Monte Carlo settings
☒	☐	For hierarchical and complex designs, identification of the appropriate level for tests and full reporting of outcomes
☒	☐	Estimates of effect sizes (e.g. Cohen's *d*, Pearson's *r*), indicating how they were calculated

Our web collection on statistics for biologists contains articles on many of the points above.

Software and code

Policy information about availability of computer code

Data collection	Raw data for cytokine quantification was collected using a VANTAstar plate reader (ELISA), a BD Luminex™ 200 instrument (Luminex), and a BD LSR Fortessa flow cytometer (CBA). Western blots and electrophoresis gels were photographed using a BioRad ChemiDoc gel imager. Images were acquired on a Leica SP8 confocal microscope.
Data analysis	We used VANTAstar MARS quantification software to compute unknown sample concentrations from manufacturer-provided standard curves. We used the built in quantification software on the Luminex instrument to calculate unknown sample values based on standard curves. We used FloJo to compute population average PE values from unknown samples, and used Microsoft Excel to compute cytokine concentrations from unknown samples based on standard curves. We used Imaris and LasX software to analyze image data. GraphPad PRISM was used to graph data. No new software was developed in this manuscript.

For manuscripts utilizing custom algorithms or software that are central to the research but not yet described in published literature, software must be made available to editors and reviewers. We strongly encourage code deposition in a community repository (e.g. GitHub). See the Nature Portfolio guidelines for submitting code & software for further information.

Data

Policy information about availability of data

All manuscripts must include a data availability statement. This statement should provide the following information, where applicable:

- Accession codes, unique identifiers, or web links for publicly available datasets
- A description of any restrictions on data availability
- For clinical datasets or third party data, please ensure that the statement adheres to our policy

All data are available in the main text or the supplementary materials.

Research involving human participants, their data, or biological material

Policy information about studies with human participants or human data. See also policy information about sex, gender (identity/presentation), and sexual orientation and race, ethnicity and racism.

Reporting on sex and gender	Not applicable.
Reporting on race, ethnicity, or other socially relevant groupings	Not applicable.
Population characteristics	Not applicable.
Recruitment	Not applicable.
Ethics oversight	Not applicable.

Note that full information on the approval of the study protocol must also be provided in the manuscript.

Field-specific reporting

Please select the one below that is the best fit for your research. If you are not sure, read the appropriate sections before making your selection.

☒ Life sciences ☐ Behavioural & social sciences ☐ Ecological, evolutionary & environmental sciences

For a reference copy of the document with all sections, see nature.com/documents/nr-reporting-summary-flat.pdf

Life sciences study design

All studies must disclose on these points even when the disclosure is negative.

Sample size	We chose sample sizes in line with current standards for in vitro and in vivo work. In vitro experiments were performed with 3 independent technical replicates per condition, and entire experiments were repeated 2-4 times (as specified in the individual figure legends) to ensure reproducibility of the phenotypes described. In vivo experiments were conducted with individual groups of 3-6 mice, and repeated 2-3 times as specified in the panels. Data presented includes all mice evaluated (across groups) for the pathogenesis and bacterial quantification experiments, and one representative replicate for the in vivo cytokine quantification experiments.
Data exclusions	Data was not excluded except in cases of clear technical error (complete loss of cells, human error, inability to resolve colony counts on a bacterial plate, equipment failure that resulted in extreme variation in temperature or humidity etc.). All technically sound data points were included.
Replication	All experiments were repeated multiple times, as indicated in the figure legends. All technically sound data was included, and we did not experience any other failures to replicate.
Randomization	Randomization was not relevant for our in vitro work: all samples were processed simultaneously through an identical pipeline (application to a contemporaneous standard curve for cytokine quantification, single camera exposure for gels/westerns, and image single-channel normalization of parameters for multi-channel confocal images). Resulting adjustments were made identically to every data-point in the resulting datasets. For our in vivo work involving mouse clinical symptom severity determinations, the same investigator who set up the groups was responsible for monitoring the experiment, so it could not be entirely randomized. However, we did recruit technicians from the mouse facility to verify our initial determinations (and they were not aware of the experimental groupings of the mice when they did so), and every effort was made to be consistent in our symptom scoring through out the experiment.
Blinding	Similar to above. Blinding was not conducted because it was not necessary when applying identical transformations to entire datasets. The primary investigator assessing mouse clinical severity symptoms was not blinded, but the confirming technicians who aided in those assessments were blinded.

Reporting for specific materials, systems and methods

We require information from authors about some types of materials, experimental systems and methods used in many studies. Here, indicate whether each material, system or method listed is relevant to your study. If you are not sure if a list item applies to your research, read the appropriate section before selecting a response.

Materials & experimental systems

n/a	Involved in the study	
☐	☒	Antibodies
☐	☒	Eukaryotic cell lines
☒	☐	Palaeontology and archaeology
☐	☒	Animals and other organisms
☒	☐	Clinical data
☒	☐	Dual use research of concern
☒	☐	Plants

Methods

n/a	Involved in the study	
☒	☐	ChIP-seq
☒	☐	Flow cytometry
☒	☐	MRI-based neuroimaging

Antibodies

Antibodies used	GAPDH, (CST#5174). Phospho-STAT1 (CST#9177S). STING (CST#13647)
Validation	All antibodies we used are commercially available from Cell Signaling Technologies (CST), and have been validated by CST for efficacy against the indicated targets. We did not perform additional validation studies.

Eukaryotic cell lines

Policy information about cell lines and Sex and Gender in Research

Cell line source(s)	We used the following cell types: RAW264.7 murine cell line from ATCC (catalog #: TIB-71), murine bone marrow derived macrophages (BMDM), and murine immortalized BMDM (iBMDM). BMDMs were isolated from WT C57BL/6J mouse femurs and tibias and differentiated for 7 days in DMEM supplemented with 50 ng/mL MCSF. iBMDMs were generously provided by Eicke Latz (University of Bonn; WT, MyD88/TRIF -/-, TLR2 -/-, and TLR4 -/-) and Kate Fitzgerald (UMass; STING -/-). TLR9 -/- iBMDMs were generated from TLR9 -/- BMDMs isolated from a male mouse and infected with the J2 virus (generously provided by Howard Young, NCI, NIH).
Authentication	No additional method was used.
Mycoplasma contamination	No additional testing was performed.
Commonly misidentified lines (See ICLAC register)	None used.

Animals and other research organisms

Policy information about studies involving animals; ARRIVE guidelines recommended for reporting animal research, and Sex and Gender in Research

Laboratory animals	We used the following mouse strains from Jackson Laboratories in this study: WT (C57BL/6J, # 000664), and TLR9 -/- (C57BL/6-Tlr9em1.1Ldm/J, #034449 – these mice were bred to homozygous KO in-house). All mice used in our experiments were 6-9 weeks old at the time of the experiment.
Wild animals	None used.
Reporting on sex	All of our experimental mouse cohorts contained equal numbers of male and female mice. We show in vivo data from the combined cohorts in each experimental group displayed.
Field-collected samples	None used.
Ethics oversight	All procedures conducted in this manuscript were approved by the NIAID IACUC committee (National Institutes of Health, Bethesda MD) on protocol LISB-3E and were performed by specifically approved investigators on the relevant protocols.

Note that full information on the approval of the study protocol must also be provided in the manuscript.

Plants

Seed stocks	None used.
Novel plant genotypes	None used.
Authentication	None used.
